# Neurosurgical interventions for patients with nasopharyngeal carcinoma: a single institution experience

**DOI:** 10.1186/1477-7819-11-227

**Published:** 2013-09-13

**Authors:** Ke Sai, Yong-gao Mou, Jing Zeng, Yan-chun Lv, Shao-yan Xi, Su Guan, Xiang-heng Zhang, Jian Wang, Chao Ke, Jian-gui Guo, Yin-sheng Chen, Zhong-ping Chen

**Affiliations:** 1 Department of Neurosurgery, Sun Yat-sen University Cancer Center, 651 Dongfeng Road East, Ghuangzhou 510060, China; 2 Department of Pathology, Sun Yat-sen University Cancer Center, Guangzhou, China; 3 Department of Imaging and Minimally Invasive Interventional Center, Sun Yat-sen University Cancer Center, Guangzhou, China; 4 Department of Experimental Research, Sun Yat-sen University Cancer Center, Guangzhou, China; 5 State Key Laboratory of Oncology in South China, Guangzhou, China

**Keywords:** Nasopharyngeal carcinoma, Radiation-induced temporal necrosis, Radiation-induced sarcoma, Multiple primary tumor

## Abstract

**Background:**

Nasopharyngeal carcinoma (NPC) is a frequent head and neck cancer in southern China and Southeast Asia. The majority of NPC patients are managed by radiation oncologists, medical oncologists and head and neck surgeons. Actually, neurosurgical interventions are warranted under specific circumstances. In this article, we described our experience as neurosurgeons in the management of NPC patients.

**Methods:**

Medical records of NPC patients who received neurosurgical procedure at Sun Yat-sen University Cancer Center were reviewed.

**Results:**

Twenty-seven patients were identified. Among 27 cases, neurosurgical procedures were performed in 18 (66.7%) with radiation-induced temporal necrosis, 2 (7.4%) with radiation-induced sarcoma, 4 (14.8%) with synchronous NPC with primary brain tumors, 2 (7.4%) with recurrent NPC involving skull base, and 1 (3.7%) with metachronous skull eosinophilic granuloma, respectively. The diagnosis is challenging in specific cases and initial misdiagnoses were found in 6 (22.2%) patients.

**Conclusions:**

For NPC patients with intracranial or skull lesions, the initial diagnosis can be occasionally difficult because of the presence or a history of NPC and related treatment. Unawareness of these entities can result in misdiagnosis and subsequent improper treatment. Neurosurgical interventions are necessary for the diagnosis and treatment for these patients.

## Background

Nasopharyngeal carcinoma (NPC) is one of the most prevalent malignancies in southern China and Southeast Asia. In a Chinese endemic area, the incidence rate of NPC reaches as high as 27.2 per 100,000 person-years in men [1]. In addition, NPC is more significantly frequent in Chinese immigrants in the United States [2]. Radiotherapy remains the mainstay treatment for NPC due to the radiosensitivity of the disease. In addition, NPC has been demonstrated to have a high response rate to multiple chemotherapeutic agents. The combination of chemotherapy with radiotherapy results in a decrease in distant metastases and improvement of locoregional control rate as well as overall survival [3]. Moreover, salvage surgery is indicated and beneficial for selected patients with local recurrent disease [4]. Therefore, the majority of patients with NPC are managed by radiation oncologists, medical oncologists, head and neck surgeons, or otolaryngologists. Occasionally, intracranial or skull lesions are found in NPC patients in the course of disease. The imaging manifestation of these lesions can be deceiving, which makes the initial diagnosis difficult. Neurosurgical procedures are therefore warranted for both diagnosis and treatment. In this article, we present a series of 27 cases in which neurosurgeons play a critical role in the management of patients with NPC.

## Methods

The current study was approved by the ethics committee of Sun Yat-sen University Cancer Center. Written informed consent was obtained from all the patients. We searched the institutional database of patients surgically treated in the Department of Neurosurgery between January 1999 and August 2012. Patients with a histological diagnosis or a history of NPC were identified and clinical records were reviewed.

## Results

A total of 27 cases were identified. Among these patients were eighteen (66.7%) with radiation-induced temporal necrosis (RITN), two (7.4%) with radiation-induced sarcoma (RIS), four (14.8%) with synchronous NPC with primary brain tumors, two (7.4%) with recurrent NPC involving the base of the skull, and one (3.7%) with metachronous skull eosinophilic granuloma (EG).

### Radiation-induced temporal necrosis

**Figure 1:**
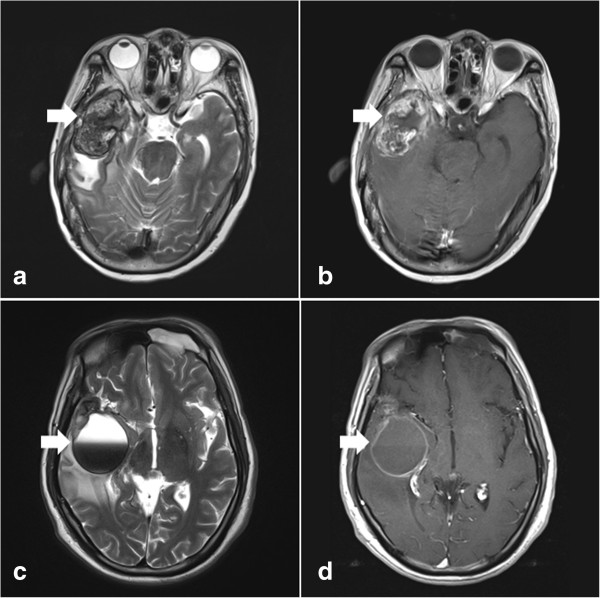
**Radiation-induced temporal necrosis (RITN) presented on magnetic resonance (MR) images.** RITN presented as a heterogeneously enhanced nodule (arrow in **a**: axial T2-weighted MR image; **b**: axial contrast-enhanced MR image) or cystic formation (arrow in **c**: axial T2-weighted MR image; **d**: axial contrast-enhanced MR image). Bleeding could be occasionally found in cystic RITN (arrowhead in **c** and **d**).

Eighteen patients (age 37 to 63 years; mean 49.6 years) with RITN after radiotherapy for NPC received neurosurgical resection (Table 1). The mean initial radiation dose was 70.5 Gray (Gy), (range 60 to 82 Gy). Four patients received repeated radiotherapy for recurrent NPC, with a dose ranging from 60 to 66 Gy. The mean latency of RITN was 6.3 years. Among the 18 patients, headache was the most common symptom, being observed in 16 patients. Fourteen patients had impaired memory, ten had vertigo, seven had neurological deficits, and one had a seizure. RITN presented on magnetic resonance (MR) images as a nodule with heterogeneous enhancement (Figure 1a and b) in nine cases and as obvious cystic formation (Figure 1c and d) in the other nine patients. Two out of nine patients with heterogeneously enhanced RITN had been initially diagnosed at the local hospital as having malignant glioma, and were then referred to our institution. Corticosteroid treatment failed and progressive increased intracranial pressure was observed in all cases. Neurosurgical resection was applied through the temporal approach. The mean postoperative follow up was 53.1 months, ranging from 5 to 114 months. One patient (patient 13) died from exhaustion 2 months after the operation and one (patient 17) died from lung cancer 40 months after treatment for RITN. One patient (patient 6) suffered from progressive radiation-induced necrosis in the contralateral temporal lobe and underwent surgery. No recurrence of necrotic lesions was observed and preoperative symptoms were improved in all 15 surviving patients.

**Table 1:** Characteristics of 18 patients with NPC with RITN

**Patient number**	**Age at diagnosis of RITN, y/Sex**	**TNM classification of NPC**	**Repeated RT for recurrence**	**Total dose, Gy**	**Latency, y**	**Side of RITN for operation**	**Follow up, mo**	**Outcome**
1	42/M	T4N3M0	No	82	9	Right	5	Improved
2	37/M	T4N1M0	No	80	7	Left	6	Improved
3	46/F	T2N1M0	No	70	7	Right	23	Improved
4	60/F	T3N1M0	No	68	8	Right	22	Improved
5	57/M	T2N0M0	No	70	5	Right	45	Improved
6	46/M	T3N0M0	Yes	70 + 65*	8	Right	53	Resection of RITN in the left side at 39 mo, improved
7	56/M	T3N1M0	No	70	7	Right	64	Improved
8	42/M	T2N0M0	No	70	7	Left	57	Improved
9	49/M	T2N0M0	No	68	8	Left	65	Improved
10	63/M	T1N0M0	No	68	6	Right	55	Improved
11	39/M	T2N0M0	Yes	68 + 60*	3	Left	99	Improved
12	37/M	T1N0M0	No	70	6	Right	87	Improved
13	58/M	T1N0M0	Yes	60 + 66*	1	Left	2	Died of exhaustion
14	59/M	T4N0M0	No	75	5	Right	114	Improved
15	47/M	T3N0M0	Yes	70 +64*	5	Right	63	Improved
16	59/M	T4N0M0	No	74	5	Left	62	Improved
17	50/M	T3N0M0	No	72	9	Left	40	Died of unrelated disease
18	45/M	T4N0M0	No	66	8	Left	94	Improved

*NPC*, nasopharyngeal carcinoma, *RITN*, radiation-induced temporal necrosis, *TNM*, tumor, node, metastasis, *y*, year, *m*, month, *Gy*, Gray, *M*, male, *F*, female, *RT*, radiotherapy, ^*^dose for repeated radiotherapy.

### Synchronous nasopharyngeal carcinoma with primary brain tumor

**Figure 2:**
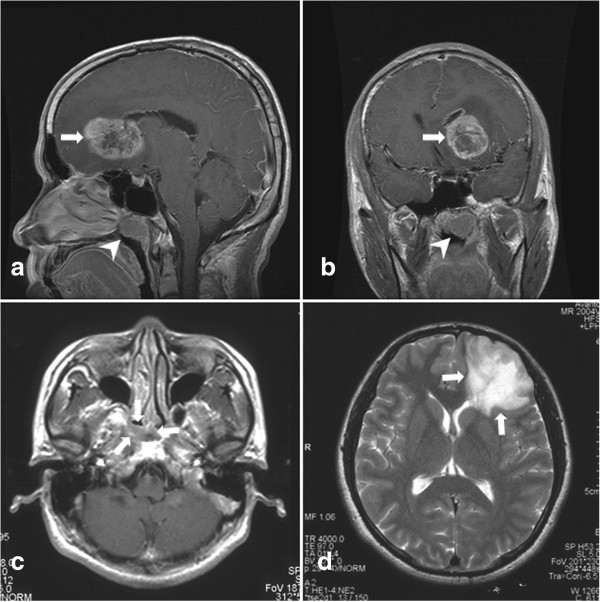
**Synchronous nasopharyngeal carcinoma (NPC) with glioma.** A 30-year old man (patient 19) suffered from headache and nasal obstruction for 6 months. On magnetic resonance (MR) images, a mass in the left lateral side and the roof of nasopharynx (arrowhead in **a** and **b**) was found. Moreover, a heterogeneously enhanced lesion was demonstrated in the left frontal lobe (arrow in **a** and **b**). Histological diagnosis was undifferentiated non-keratinizing carcinoma for the mass in the nasopharynx, and glioblastoma multiforme for the left frontal lobe lesion. A 37-year old man (patient 20) presented with epistaxis for 2 months. The MR scan demonstrated a mass in the right lateral side and the roof of the nasopharynx (arrows in **c**: axial contrast-enhanced MR). In addition, a left frontal lesion with hypodensity on T1-weighted images and hyperintensity on T2-weighted images (arrows in **d**: axial T2-weighted MR), but without gadolinium enhancement, was synchronously found on the MR scan. Endoscopic biopsy revealed a non-keratinizing NPC. Subtotal resection of the left frontal lesion was achieved and postoperative histological examination established the diagnosis of World Health Organization grade II astrocytoma.

**Figure 3:**
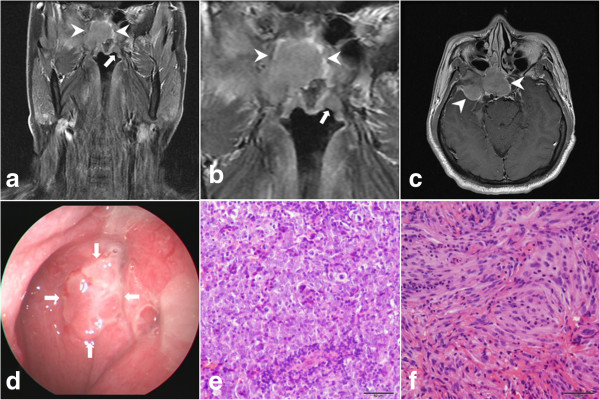
**Synchronous nasopharyngeal carcinoma (NPC) with meningioma in the anterior and middle base of the skull.** A 45-year old man (patient 21) presented with hearing impairment in the right ear and dizziness for 10 days. The magnetic resonance (MR) scan demonstrated a homogeneously enhanced mass involving the sphenoidal sinus, anterior skull base and right middle skull base (arrow heads in **a**, **b** and **c**: contrast-enhanced MR). A lesion in the left lateral side of the nasopharynx (arrows in **a** and **b**: coronal contrast-enhanced MR) was also identified. Endoscopic biopsy of the nasopharyngeal lesion (arrows in **d**: endoscopic view) and sphenoidal mass was performed. Histological examination revealed the diagnosis of NPC with strong positive immunohistochemisty staining for P63 and CK in the nasopharynx (**e**: hematoxylin and eosin staining, ×200) and meningioma immunopositive for epithelial membrane antigen (EMA) and vimentin but negative for CK and Epstein-Barr virus-encoded RNA *in situ* hybridization (EBER ISH) in the sphenoidal sinus (**f**: hematoxylin and eosin staining, ×200).

NPC was simultaneously diagnosed with glioma in two patients (patients 19 and 20, Table 2, Figure 2) and with meningioma involving the anterior skull base in two patients (patients 21 and 22, Table 2, Figure 3), respectively. One patient (patient 20) had a family history of cancer. His elderly sister was also suffered from NPC and one of his children was diagnosed with acute lymphoblastic leukemia. NPC was detected in all four patients by nasopharyngoscopy and histologically confirmed by biopsy. MR scanning revealed the coexistence of an intracranial mass. Patient 19 was initially diagnosed as having NPC with intracranial metastasis and patient 21 as having NPC with invasion of the skull base; both were diagnosed at the local hospital. All four patients received microneurosurgery followed by treatment for NPC. Total removal was achieved in two patients with meningioma and subtotal removal in two with glioma. In all four patients, NPC was treated with radiotherapy with or without chemotherapy. Glioma in two patients was also treated with radiation and adjuvant chemotherapy. The follow up ranged from 10 to 79 months. Two patients with meningioma had no recurrence of NPC or intracranial tumor. One patient (patient 19) with glioblastoma multiforme (GBM) died 10 months after the initial diagnosis because of ventricle dissemination. The other patient with World Health Organization (WHO) grade II astrocytoma (patient 20) experienced malignant transformation of the tumor and recurrence.

**Table 2:** Characteristics of patients with synchronous NPC and brain tumor

**Patient number**	**Age at diagnosis, y/Sex**	**TNM classification of NPC**	**Location/type of synchronous brain tumor**	**Treatment for NPC**	**Treatment for brain tumor**	**Follow up, mo**	**Outcome**
19	30/M	T3N2M0	Left frontal lobe/GBM	Chemoradiotherapy, chemotherapy	Subtotal removal, radiotherapy, chemotherapy	10	Died of glioma at 10 months
20	37/M	T1N0M0	Left frontal lobe/WHO grade II astrocytoma	Radiotherapy	Subtotal removal, radiotherapy, chemotherapy	82	Survival with recurrent glioma
21	45/M	T2N0M0	Sphenoidal sinus, anterior and middle skull base/meningioma	Radiotherapy	Total removal	52	Survival without recurrence
22	68/M	T4N2M0	Anterior skull base/meningioma	Chemoradiotherapy, chemotherapy	Total removal	24	Survival without recurrence

*NPC*, nasopharyngeal carcinoma, *TNM*, tumor, node, metastasis, *M*, male *mo*, month, *GBM*, glioblastoma multiforme, *WHO*, World Health Organization.

### Radiation-induced sarcoma

**Figure 4:**
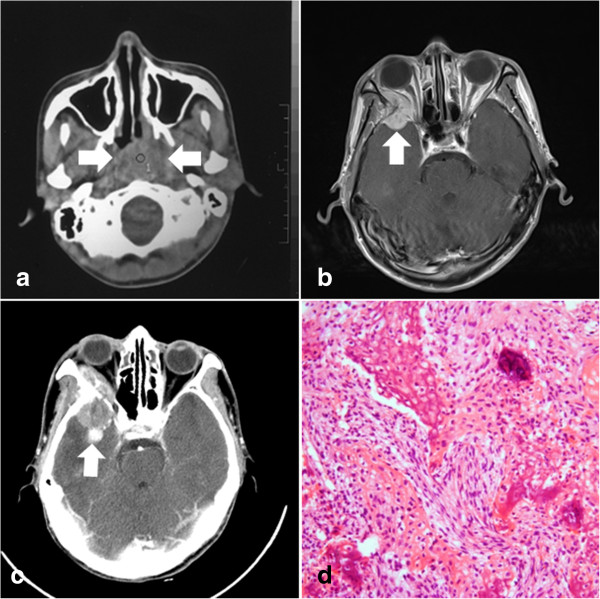
**Radiation-induced sarcoma in a patient with nasopharyngeal carcinoma (NPC).** A 25-year-old man (patient 23) had undergone irradiation with a total dose of 70 Gy for undifferentiated NPC (arrow in **a**: computed tomography (CT) scan). Approximately 10 years after radiotherapy, he presented with pain in the right temporal region and exophthalmus of his right eye. Magnetic resonance (MR) imaging demonstrated a contrast-enhanced lesion in the right temporal region with intracranial and orbital invasion (arrow in **b**: axial contrast-enhanced MR image).One month later, the preoperative CT scan revealed significant enlargement of the lesion (arrow in **c**). Subtotal resection was performed and postoperative histological examination showed nests and sheets of pleomorphic malignant tumor cells with scattered osteoid formation, consistent with an osteosarcoma (**d**: hematoxylin and eosin staining, ×100).

Two cases of RIS (Table 3) were identified in this series according to the diagnosis criteria defined by Cahan [5]. Both patients were men. The radiation dose was 70 Gy for NPC in patient 23 and 68 Gy in patient 24. The latency of RIS after radiotherapy was 119 months for patient 23 (Figure 4) and 117 months for patient 24. Subtotal resection was achieved because of the extensive invasion to adjacent structures in the skull base. Post-operative radiotherapy was administered for both patients and chemotherapy was also given for patient 24. Patient 23 was followed up for 13 months and local recurrence was observed 7 months after initial diagnosis. The patient was still alive but refused any further treatment. Patient 24 died of recurrent RIS 6 months after initial diagnosis.

**Table 3:** Characteristics of five NPC patients with indications for neurosurgical procedures other than RITN and synchronous brain tumor

**Patient number**	**Age at diagnosis of NPC, y/Sex**	**TNM classification of NPC**	**Indication for neurosurgical intervention**	**Months between diagnosis**	**Treatment**	**Follow up, mo**	**Outcome**
23	25/M	T4N1M0	Radiation-induced sarcoma	119	Subtotal removal, radiotherapy	13	Survival with recurrence
24	42/M	T2N1M0	Radiation-induced sarcoma	117	Subtotal removal, radiotherapy, chemotherapy	6	died of local recurrence
25	39/F	T1N2M0	NPC recurrence in the cerebellopontine angle	45	Total removal, chemotherapy, radiotherapy	23	Survival without recurrence
26	55/M	T4N1M0	Mastoid recurrence of NPC	21	Biopsy, chemotherapy, radiotherapy	37	Survival without recurrence
27	44/M	T2N2M0	Metachronous skull eosinophilic granuloma	14	Resection of frontal lesion	7	Stable

NPC, nasopharyngeal carcinoma: RITN, radiation-induced temporal necrosis; M, male; y, year; mo, month.

### Recurrent nasopharyngeal carcinoma involving the skull base

**Figure 5:**
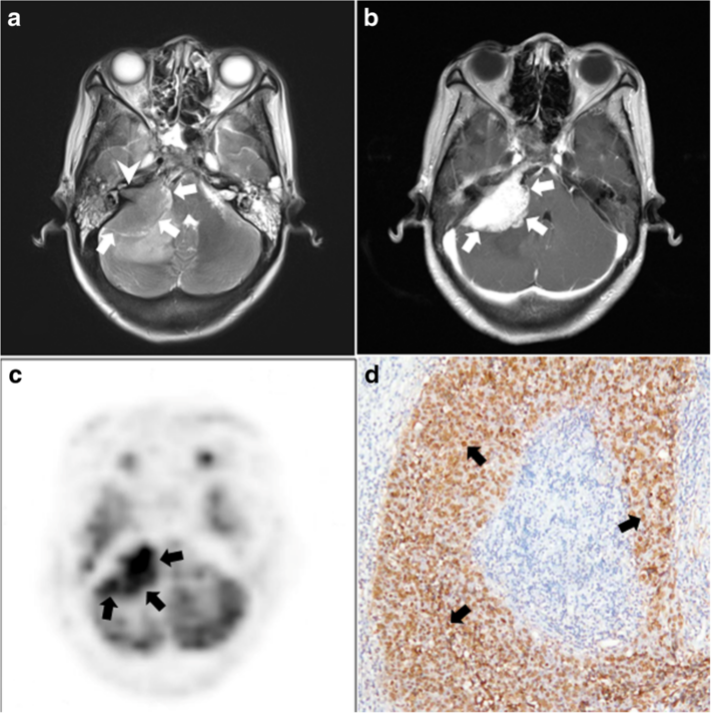
**Recurrent nasopharyngeal carcinoma (NPC) in the cerebellopontine angle (CPA).** A homogeneously enhanced lesion with clear demarcation in the right CPA (arrows in **a**: T2-weighted magnetic resonance (MR) image and arrows in **b**: contrast-enhanced MR) was found (patient 25) 4 years after irradiation for NPC. Enlargement of the right internal meatus (arrow head in **a**: T2-weighted MR image) was also demonstrated. The lesion (arrows in **c**: ^18^F-fluorodeoxyglucose positron emission tomography (^18^F-FDG PET)) and the neck lymph node had increased uptake of ^18^F-FDG. Postoperative histological examination revealed an undifferentiated non-keratinizing carcinoma immunopositive for CK and P63 and positive for Epstein-Barr virus-encoded RNA *in situ* hybridization (EBERs ISH) (arrows in **d**: positive EBERs ISH in tumor cell nuclei, ×100).

**Figure 6:**
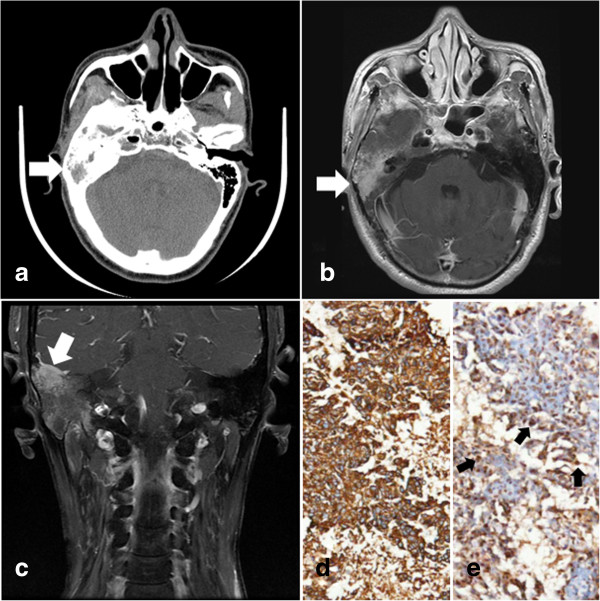
**Recurrent nasopharyngeal carcinoma (NPC) in the mastoid antrum.** The imaging follow up with computed tomography (CT) demonstrated a soft tissue mass (arrow in **a**: CT scan) in the right mastoid antrum of patient 26, who had been diagnosed and treated for NPC 2 years before. Magnetic resonance (MR) imaging showed an enhanced lesion in the right mastoid antrum (arrow in **b** and **c**: contrast-enhanced MR). Histological examination after biopsy revealed an undifferentiated non-keratinizing carcinoma, consistent with NPC (**d**: strong cytoplasmic staining of CK in tumor cells, ×100; arrows in **e**: positive Epstein-Barr virus-encoded RNA *in situ* hybridization (EBERs ISH) in tumor cell nuclei, ×100).

We reported two cases with recurrent NPC involving the base of the skull, with one in the cerebellopontine angle (CPA) and the other in the mastoid antrum (Table 3). A 43-year-old female patient (patient 25, Figure 5) manifested with double vision and right facial numbness 4 years after radiotherapy and chemotherapy for NPC. Physical examination revealed palsy of the right abducens and facial nerve, right trigeminal-distribution facial hypesthesia, and ataxia. MR demonstrated a marked contrast-enhanced lesion in the right CPA with enlargement of the right internal auditory meatus. The lesion was radiologically diagnosed as an acoustic neuroma. Because the growth pattern was not typical of an acoustic neuroma, ^18^F-fluorodeoxyglucose positron emission tomography (^18^F -FDG PET) was performed, which demonstrated an increased level of ^18^F-FDG in the lesion and in the neck lymph node. Invasion of NPC into the base of the skull was therefore suspected. The patient underwent total removal of the mass through the retrosigmoid approach and postoperative histological examination revealed undifferentiated NPC. Postoperative radiotherapy and chemotherapy was administered for this patient. The patient was followed up for 23 months and no recurrence was found. Another 57-year-old male patient (patient 26, Figure 6) had previous treatment of NPC 2 years before imaging follow up and a lesion was found in the right mastoid antrum. The lesion had been treated at the local hospital as mastoiditis but no improvement was observed. ^18^F-FDG PET was performed on this patient at our institution and demonstrated increased uptake of ^18^F-FDG. Radiation-induced temporal bone malignancy was suspected. Biopsy of the mastoid lesion was performed. A histological diagnosis of NPC was confirmed. The patient then received radiotherapy for the mastoid recurrence of NPC. The patient is still alive after 37-months of follow up.

### Metachronous skull eosinophilic granuloma

**Figure 7:**
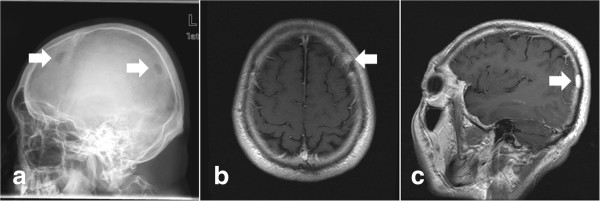
**Metachronous skull eosinophilic granuloma.** Patient 27 presented with a tender palpable frontal mass 16 months after he had been treated for nasopharangeal carcinoma. Radiography of the skull demonstrated two bony erosions (arrows in **a**) in his frontal and occipital bone. The lesions were contrast-enhanced on the magnetic resonance image (arrow in **b** and **c**). Histological examination demonstrated Langerhans cells and eosinophils with positive immunohistochemistry for S100 and CD1a.

A 45-year-old male patient (patient 27, Table 3, Figure 7) had a tender palpable mass in his frontal bone and pain for 1 month. Radiography of the skull demonstrated two bony erosions in his frontal and occipital bone. These two lesions were contrast-enhanced on the MR image. Bone scintigraphy using Tc-99 m methylene diphosphonate (MDP) revealed increased uptake of the nuclide by the frontal lesion. The patient had been diagnosed with NPC 16 months before and had received chemotherapy and radiation. No osteolytic lesion was detected in the skull by the nuclide bone scan at that time. The patient was initially diagnosed as having skull metastasis from NPC. History of cancer was found in his family; his maternal uncle had been diagnosed with NPC, his father died of renal cancer and his uncle died of liver cancer. On admission, no recurrence of NPC was found. Resection of the frontal lesion was performed and the postoperative histological diagnosis was EG.

## Discussion

Although uncommon in western countries, NPC is a frequent head and neck cancer in southern China and Southeast Asia. NPC is sometimes referred to as Cantonese cancer because the incidence of NPC in a specific region in Guangdong province is 25 times higher than that in the rest of the world. Chinese emigrants and their family members still have a high risk of NPC. The striking regional distribution has been found to be associated with the extensive consumption of salted fish and preserved vegetables in the endemic areas [6]. More importantly, a recent genome-wide association study revealed the association of NPC with the HLA-A region, suggesting the influence of genetic factors in the etiology of NPC [7]. In our institution, more than 2,000 NPC patients were treated every year. The majority of these patients were managed by radiation oncologists, medical oncologists and head and neck surgeons. With a large NPC patient population, we had the opportunity to identify a series of 27 cases, for which neurosurgical interventions were necessary. The entities presented in the current article included (1) late stage RITN; (2) RIS; (3) simultaneously diagnosed NPC and brain tumors; (4) recurrent NPC with the involvement of the skull base; and (5) metachronous skull EG. Although uncommon, these cases are of great clinical significance. The presence or history of NPC or the treatment-related complications makes it difficult initially to accurately diagnose the intracranial or skull lesions in specific cases. Unawareness of these entities can result in delayed diagnosis, or even misdiagnosis and subsequent improper treatment. Among twenty-seven cases, six (22%) were initially misdiagnosed. Neurosurgical interventions are indispensible for the accurate diagnosis and treatment of intracranial or skull lesions in these NPC patients.

RITN is a severe late sequel for NPC patients after radiotherapy and composes the major disease entity in our series, accounting for 66.7% of all cases. The temporal lobes are anatomically adjacent to the nasopharyngeal cavity and are therefore inevitably delineated in the irradiation field. The development of RITN has been suggested to be associated with the total dose of the external beam radiation, the fraction schedule, and possibly with the chemotherapy administration [8,9]. Radiation-induced progressive damage to vessel endothelium and migration of inflammatory cells are proposed to be the mechanisms underlying RITN [10]. On MR images, the early stage of RITN manifests as patchy or nodular enhancement with extensive edema. In the late stage, cystic formation or heterogeneous necrotic enhancement can be observed. Cystic RITN is usually not difficult to diagnose, and should be distinguished from a temporal abscess. But as for heterogeneously enhanced temporal lesions in irradiated patients with NPC, it is sometimes challenging to establish the accurate diagnosis based on MR alone. Without consideration of the history of irradiated NPC, a radiologist or a neurosurgeon may diagnose primary malignant brain tumors in these cases. In our series, two of nine patients with heterogeneously enhanced RITN had been radiologically diagnosed as having malignant glioma. ^18^F-FDG PET has been employed complementary to MR imaging, with the assumption of its superiority to distinguish necrosis from an actively proliferating malignant tumor, by evaluating the metabolic level in the lesion. However, we and other groups found that ^18^F-FDG can be significantly taken up by some necrotic lesions, which may lead to misdiagnosis of malignancy [11,12]. The metabolic burst of inflammatory cells in necrosis has been suggested to account for the false positive findings in ^18^F-FDG PET [13]. As for treatment, the administration of steroids is most frequently used in the management of early stage RITN because steroids are capable of relieving the inflammatory reaction and reducing cerebral edema. However, recent findings suggested that steroid therapy temporarily results in symptomatic relief but fails to change the biological process of RITN, and can also cause serious complications [14]. Recently, a prospective trial demonstrated that treatment with bevacizumab, a monoclonal antibody against vascular endothelial growth factor, achieves a significant improvement in symptoms and reversal of disease on imaging in the majority of patients with radiation-induced brain necrosis [15]. Although this result is exciting, all patients enrolled in this trial were at an early stage of brain necrosis. Neurosurgical intervention is necessary when RITN is at a late stage with significant mass effect after the failure of conservative therapy. The prognosis of RITN after surgery is favorable. Resection of necrotic lesions not only immediately relieves the potentially life-threatening increased intracranial pressure, but also is likely to be effective in promptly controlling brain edema and attenuating the progression of necrosis by removing its inflammatory components [16].

In addition to RITN, RIS is an uncommon but severe complication of radiotherapy in NPC patients. In general, the estimated incidence of RIS ranges from 0.03% to 0.30% [17,18]. For NPC, epidemiological data are limited but two recent studies demonstrated that RIS accounts for less than 0.2% of patients [19,20]. The development of RIS is suggested to be associated with the previous radiation dose and the genetic susceptibility of the specific population [21-23]. RIS appears to be one of the most intractable diseases and treatment is particularly challenging. Surgical resection is still the preferred option for RIS and total removal has been shown to be the most significant prognostic factor. Patel *et al*. demonstrated that the median overall survival was 47 months for RIS patients with gross total resection compared to only 27 months for those with incomplete removal [24]. Nevertheless, RIS in the head and neck is often hard to remove completely because of its deep-seated location around the skull base and the proximity to vital neural and vascular structures. Without a negative surgical margin, local growth and recurrence are frequently observed, and that is one of the possible reasons why RIS in the head and neck has a worse outcome than its counterpart in some other organs, such as the extremities [25]. In patients 23 and 24, subtotal resection only was achieved because of the extensive invasion of RIS to intracranial, orbital and subtemporal structures. The role of adjuvant treatment such as radiotherapy and chemotherapy for RIS is controversial. Although radiotherapy has a definite role in the management of primary sarcoma, it has seldom been recommended for RIS [26,27]. The high cumulative radiation dose to adjacent organs, inefficacy of radiation in RIS and the risk of inducing another malignancy are the major concerns for re-irradiation. But in a report by Tan, RIS demonstrated a significant response to repeated radiation. The RIS patient described in that report achieved five-year disease-free survival after preoperative irradiation followed by *en bloc* resection of the tumor [28]. Similarly, the role of chemotherapy for RIS is hard to assess. A few authors have adopted regimens from primary sarcomas and administered these for treatment of RIS. Efficacy of chemotherapy has been observed in some studies but was not confirmed by others [24,29-31]. Therefore, the aggressive removal achieved by neurosurgeons is still the mainstay for the treatment of RIS in NPC patients, and the efficacy of radiotherapy and chemotherapy requires further investigation.

Coexistence of NPC and another type of tumor, such as breast cancer, renal cancer and lymphoma, has been episodically reported [32-34]. But synchronous NPC with primary brain tumors has not been described in English literature. We identified four such cases, among which two NPC patients were simultaneously diagnosed with glioma and two with meningioma involving the anterior skull base. The development of multiple primary tumors has been suggested to be associated with the shared risk factors of different tumor types and tumor predisposition in individuals [35]. Epstein-Barr virus infection, salt-preserved fish consumption and smoking are well-known risk factors for NPC, but these have not been confirmed as responsible for the etiology of glioma and meningioma [36]. The predisposition to tumors probably accounts for the synchronousness in these four cases. As an example, in the case of patient 20, his sister suffered from NPC and his son was diagnosed with leukemia. We speculated that specific genetic alterations made him susceptible to NPC and glioma. The initial diagnosis was elusive for these cases because it is complicated by the presence of NPC. In patient 19, the primary brain tumor was initially diagnosed as a metastasis from the NPC, and the meningioma in patient 21 was considered to be due to invasion of NPC into the base of the skull. Without having performed histological examination of tissues from the neurosurgical resection, these patients could have been treated as late-stage NPC with radiotherapy and chemotherapy. If so, these patients would have suffered from toxicity due to inappropriate treatment.

In the current article, we reported two cases of recurrent NPC with the involvement of the base of the skull, one of which was found in the CPA and the other in the mastoid antrum. NPC has a predilection to involve the skull base because of its anatomical features. The regions in the skull base most frequently involved with NPC are the pterygoid process, the base of the sphenoid bone and the petrous apex [37]. However, spread to the CPA is uncommon, with an estimated incidence of approximately 0.05% for primary disease and 1.0% for recurrent cases [38]. Direct extension via the jugular foramen, hematogenous or lymphatic metastasis, and dissemination through cerebrospinal fluid are suggested as possible mechanisms [39-41]. In addition, minimization of the radiation dose to the brainstem in order to protect it from radiation-induced injury during radiotherapy for NPC may place the CPA in the low-dose or high-dose gradient area. Therefore, the undertreated microscopic foci may progress to a symptomatic lesion in the CPA. Early diagnosis of recurrent NPC involving the CPA is difficult because the initial symptom of sensorineural deafness or headache in these patients can be assumed to be the sequel of irradiation and impaired function of the facial nerve may be considered to be Bell’s palsy. Even with imaging examinations, it is challenging to diagnose the disease accurately, especially when there is no recurrence in the nasopharynx. Recurrent NPC in the CPA can extend into the internal auditory meatus masquerading as an acoustic neuroma [42]. In patient 25, the recurrent disease was radiologically diagnosed as an acoustic neuroma. We employed ^18^F-FDG PET to facilitate the diagnosis. The significantly increased uptake of ^18^F-FDG by the lesion and neck lymph node supported the diagnosis of a recurrence in CPA. In general, chemotherapy and re-irradiation are recommended for recurrent NPC. But in this case, neurosurgical removal was first performed because of the space-occupying effect of the lesion. After surgery, this patient was treated with radiotherapy and chemotherapy.

Mastoid recurrence of NPC is extremely unusual. Only two cases have previously been reported [43]. Although the mechanism of how NPC cells reach the mastoid antrum remains unclear, the eustachian tube is extrapolated to be the route. Mastoiditis and a radiation-induced temporal bone tumor are among the differential diagnoses. Because the treatment and prognosis of the three diseases mentioned above are totally different, the histological examination is critical. In patient 26, we performed biopsy of the lesion in the mastoid antrum. When analysis of the intra-operative frozen section suggested a diagnosis of NPC, we terminated the surgical procedure because extensive resection is not necessary for NPC, in contrast with radiation-induced tumors, and may potentially result in impairment of the facial nerve. The patient then received chemotherapy followed by re-irradiation and no recurrence has been observed.

The association between Langerhans cell histiocytosis (LCH) and different neoplasms other than NPC has been frequently reported. Howarth *et al*. reported the largest series of 27 cases of coexisting LCH with other neoplasms, accounting for 8.6% of all LCH patients [44]. Lung cancer, hematological malignancy and endocrine tumor were found in the group of patients. In addition, synchronous or metachronous diagnosis of LCH with Hodgkin’s disease has been repeatedly described in the literature [45]. However, EG in an adult after treatment for NPC has not previously been described. Common etiological factors, altered immunity and genetic predisposition were postulated as mechanisms underlying the association between LCH and other neoplasms. For patient 27, genetic alteration suggested by the cancer predisposition in his family, and immune dysfunction resulting from NPC and its related treatment possibly accounted for the metachronous tumors. The diagnosis was initially elusive in this case. Multiple bone erosions in an adult patient with a history of NPC was reminiscent of metastasis [46]. The histological diagnosis established by neurosurgical resection was critical for this patient because the treatment strategy and prognosis are totally different for LCH and NPC bone metastasis.

## Conclusions

In the current retrospective study, we reported 27 NPC patients treated in the neurosurgical department in an NPC endemic region. These cases are uncommon but clinically important. The initial diagnosis of intracranial or skull lesions in NPC patients can be complicated by the presence or history of the disease or treatment-related complications. Initial misdiagnosis and delayed diagnosis was found in specific patients. Medical practitioners who manage NPC patients should be aware of entities reported in the current article in order to avoid misdiagnosis and improper treatment. In these cases, neurosurgical interventions are necessary for the establishment of histological diagnosis and planning of treatment.

## Abbreviations

CPA: cerebellopontine angle; CT: computed tomography; EBERs ISH: Epstein-Barr virus-encoded RNA *in situ* hybridization; EG: eosinophilic granuloma; EMA: epithelial membrane antigen; F-FDG PET: ^18^F-fluorodeoxyglucose positron emission tomography; GBM: glioblastoma multiforme; LCH: Langerhans cell histiocytosis; MDP: methylene diphosphonate; MR: magnetic resonance; NPC: nasopharyngeal carcinoma; RIS: radiation-induced sarcoma; RITN: radiation-induced temporal necrosis; WHO: World Health Organization.

## Competing interests

The authors declare that they have no competing interests in this study.

## Authors’ contributions

KS and ZC contributed to the conception, analysis of the data and manuscript drafting. ZC, YM and JW are senior neurosurgeons who performed neurosurgical procedures for these patients. YL analyzed all of the imaging data. SX and JZ carried out the histopathological analysis. SG, XZ, CK, JG and YC collected the data and followed up patients. All authors read and approved the final manuscript.
